# Setting directions for capacity building in primary health care: a survey of a research network

**DOI:** 10.1186/1471-2296-7-8

**Published:** 2006-02-09

**Authors:** Karin Ried, Elizabeth A Farmer, Kathryn M Weston

**Affiliations:** 1 Primary Health Care Research Evaluation and Development (PHCRED) Program, Department of General Practice, Flinders University, GPO Box 2100, Adelaide SA 5001, Australia

## Abstract

**Background:**

The South Australian Research Network 'SARNet' aims to build research capacity in primary health care, as part of a national government-funded strategy to integrate research into clinical practice. Internationally, research networks have been a fundamental part of research culture change, and a variety of network models exist. The 'SARNet' model uses a whole system, multidisciplinary approach to capacity building and supports individuals and groups. We undertook a descriptive baseline survey in order to understand the background and needs of SARNet members and to tailor network activities towards those needs.

**Methods:**

A questionnaire survey, assessing members' professional background, research experience, and interest in research development and training, was sent to all members who joined the network in its first year. The visual 'research spider' tool was used to ascertain members' experience in ten core research skills, as well as their interest in developing these skills. Individuals were asked to classify themselves into one of four categories of researchers, based on previous research experience. These self-assessment categories ranged from non-participant to academic.

**Results:**

Network membership was diverse. Of the 89 survey participants, 55% were general practitioners or allied health professionals. Overall, most survey respondents indicated little to moderate experience in 7 out of the 10 skills depicted in the 'research spider'. In comparison, respondents were generally highly interested in developing their research skills in all areas. Respondents' research skills correlated significantly with their self-assessed category of research participation (Spearman rank correlation, r = 0.82, p < 0.0005). Correlations between research category and publication record (Gamma association, γ = 0.53, p < 0.0005) or funding record (Gamma association, γ = 0.62, p < 0.0005) supported the internal validity of the survey instrument.

**Conclusion:**

Literature describing evaluation of the impact of networks is scarce. Our survey questionnaire could provide a useful instrument for evaluation of both networks and capacity building initiatives. The survey including the 'research spider' tool provided valuable information about members' needs and interest in strategies to develop their research skills. Initial needs analyses as well as on-going evaluation of network activities are important to include into the business plans of research networks, in order to ensure the network's effectiveness and support of its membership.

## Background

There is increasing international interest in research capacity building in primary health care.

In 2000, the Australian Government announced a $50 m research capacity building initiative, known as the Primary Health Care Research Evaluation and Development (PHCRED) program. The aim of the program is to develop the research and evaluation skills of primary health care professionals and to improve the uptake of evidence into clinical practice. From 2000–2005, Australian University Departments of General Practice and Rural Health received funding of about $200,000 each per annum to engage in locally relevant capacity building strategies. Each Department was able to design their strategies as they wished, subject to approval of a strategic plan by the funding body.

The development of networks as a strategy to increase research capacity has been a growing focus of attention in the international literature in the last decade [1]. For example, in the UK, research networks commenced in 1991. At first, there was an opportunistic and uncoordinated approach to network development based on the enthusiasm and vision of individuals and groups. However, concurrent Scottish initiatives, focussing mainly on the quality of health services research, added weight to the push for capacity building. Networks grew strongly and by 1996–7 there were 23 active in the UK [2]. Recently, over 40 primary care research networks have joined together under the umbrella of the UK Federation of Primary Care Research Organisations [3]. The main objectives of the Federation include promotion of research in clinical practice and providing access to and dissemination of information on potential research. The Federation also aims to foster and facilitate collaboration, research training opportunities, research funding and academic advice, and to encourage participation of practitioners in research activities. Through opportunities for training and promotion of the use of research, the Federation hopes to advance change in the research culture of primary care.

In Australia, the Flinders University PHCRED program began research capacity building by developing a conceptual model upon which to build its strategies with defined purpose [4]. The Flinders model defined research capacity building as a whole system approach, which promotes participation of new researchers through to more experienced practitioners. The model also emphasises accommodating diversity amongst primary health care practitioners, enabling collaboration and reducing barriers to participation, as well as facilitating and promoting mentoring and networking. Building on the model, the Flinders PHCRED team developed a multidisciplinary collaborative research network aimed at participants with diversity of skills and experiences, and called it the South Australian Research Network for primary health care, shortened to 'SARNet' [5].

The aim of SARNet is to expand the pool of research-aware and research-oriented primary health care practitioners in South Australia and interstate. [See Additional File 1] for SARNet's aims and objectives. SARNet provides a practical strategy to build capacity at all levels of research and evaluation experience. The levels of research skills and experience can be divided in four, simple, logical categories of research involvement: Non-participants, participants, research managers and trainers, and academics [see Additional File 2].

SARNet was launched in November 2002 attracting 229 members in the first year. To understand the background and skills of the membership and to tailor SARNet services to members' needs, a baseline survey was sent to all members who joined between November 2002 and December 2003. Here, we report the results of the SARNet baseline survey, which provided information to assist the Flinders PHCRED Program in developing capacity building activities relevant to network members.

## Methods

### Study design

We designed a survey questionnaire to assess network members' research experience and interest in developing further skills. The questionnaire sought information in five main areas:

1. personal and professional background,

2. current level of participation in research,

3. level of experience in ten specific research skills,

4. publication and funding record,

5. interest in a range of potential opportunities including skills development, training, on-line resources, and networking activities.

The level of participation in research, assessed in part two of the survey, was divided into four categories, as suggested by Farmer & Weston [4], i.e. non-participants (little or no previous experience in research); participants (as part of a research team); managers/trainers (either leading research, or in formal training to do so); and academics (with, or leading toward, a doctorate).

Part three of the survey sought to ascertain the level of research experience using the visual 'research spider', a simple instrument for self-evaluation of knowledge and skills in ten core areas [6]. These include 'writing a research protocol', using quantitative research methods', 'publishing research', 'finding relevant literature', and 'applying for research funding' [all ten core areas are displayed in Figure 2]. In each area, the level of experience was measured on a five-point scale, from 1 (no experience) to 5 (high experience).

**Figure 1:**
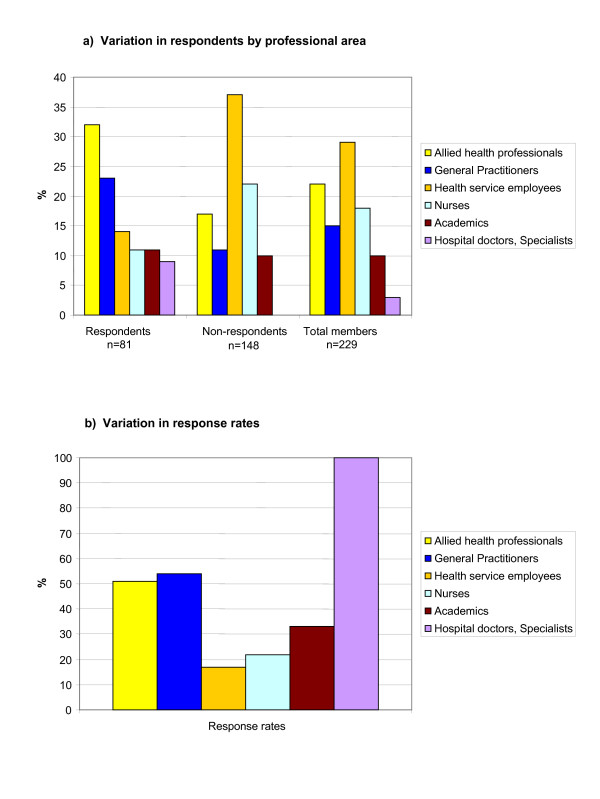
**Professional area of SARNet members 2002/03**. **a) Variation in respondents by professional area**. **b) Variation in response rates by professional area**. Figure 1a illustrates the professional areas of survey respondents in comparison to non-respondents, and all members. SARNet members were from diverse primary health care professions. The majority of respondents (55%) worked in allied health and general practice. 'Allied health' included consumer/community health, aged care, child, youth and women's health, chronic illness, mental health, nutrition, occupational therapy, pharmacy. General practice included divisional staff. Other professional areas were nursing, health services including coordinators, managers and information technologists, medical specialists, and academics. Figure 1b summarises the variation in response rates of each professional group.

**Figure 2:**
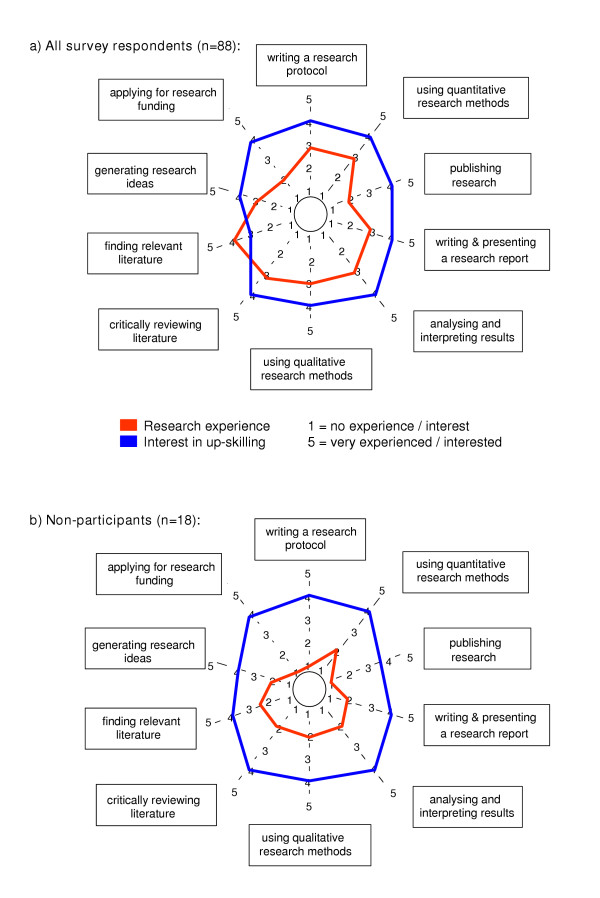
**Median research experience and interest in up-skilling**. **a) of all survey respondents (n = 88)**. **b) of non-participants (research category 1) (n = 18)**. The 'research spider' [6] was used to collect information on individual research experience and interest in research skill development. The level of experience or interest in developing a particular skill were measured using a five point scale ranging from 1 (no experience or interest) to 5 (high experience or interest). The red line depicts the median score of respondents' research experience. The blue line depicts the median score of respondents' interest in developing research skills. While the interest (blue line) in up-skilling of non-participants (b) matches the interest level of all survey respondents (a), the level of research experience (red lines) is lower for non-participants (b versus a) in all ten skill areas.

The fifth part of the questionnaire assessed members' interest in development of research skills, training, resources and network activities. The level of interest in developing skills in ten core areas was ascertained using the 'research spider' a second time in the survey. Suggested training included events such as short courses (2–3 hrs), advanced courses (1–2 days), writing workshops (8 × 1 hr), and state-wide conferences. Suggested resources included a network website linking to training opportunities, evidence-based practice resources, and primary health care journals. Suggested networking activities comprised journal clubs, mentoring activities, and communication facilities such as an email list server and an online bulletin/discussion board. In each area, the level of interest was measured on a five-point scale, from 1 (no interest) to 5 (high interest).

Primary health care professionals, students and consumers who joined the network in its first year were invited to participate in the survey. All 229 members were posted a survey questionnaire, information about the study, and a reply paid envelope within 2 weeks of joining the network. An individual reminder email was sent within 2 weeks of posting the questionnaire. A general reminder email was sent to all members 2 months before the final date of data collection. If requested, a second questionnaire was posted to the individual. No additional incentives other than access to and regular information about all network activities were given for participation in the study. Completed surveys were returned anonymously.

A copy of the survey questionnaire can be obtained from the corresponding author. The study was approved by the Human Research Ethics Committee at Flinders University.

### Data analysis

Open ended questions (e.g. "Comment on the reasons for joining SARNet") or questions allowing multiple answers (e.g. "List any awards and research funding received") were coded into meaningful categories and analysed accordingly. We used the statistical software package SPSS 12.0 to analyse the data both ungrouped and according to the four self-assessed research participation categories. Median scores of all survey participants for each of the ten research skills depicted in the 'research spider' were calculated and compared to the median scores for the interest in up-skilling. Additionally, grouped median scores of all ten research skills were analysed by the four research categories. Correlations between research categories and research skills were ascertained by the 'Spearman rank test'. The 'Goodman and Kruskel gamma measure of association' was used to determine the significance of the correlation between publication as well as funding records and self-assessed research categories.

## Results

### Response rates and member demographics

A total of 89 members (40% of all first year members) participated in the survey. More female health professionals (74%) than male health professionals joined SARNet in 2002/03, and accordingly represented the majority of respondents (76%). The median age of all respondents was 40, ranging from 21 to 62 years. The majority of survey respondents (75%) were residents of metropolitan South Australia, the remainder representing rural South Australia and interstate. Figure 1 and Table 1 summarise the professional or study areas of the survey respondents in comparison to non-respondents. Allied health professionals and GPs represented the majority (55%) of survey respondents.

**Table 1:** SARNet member demographics (2002/03)

	**Number (Percentage)**			
**Demographics**	**Respondents**	**Non-respondents**	**Total members**	**Response rate**

	*n (%)*	*n (%)*	*n (%)*	*%*
**Profession**				
Total number *	*81 (100)*	*148 (100)*	*229 (100)*	*35*
Allied health professionals	26 (32)	25 (17)	51 (22)	51
General Practitioners, Divisional staff	19 (23)	16 (11)	35 (13)	54
Health service employees	11 (14)	55 (37)	66 (29)	17
Nurses	9 (11)	32 (22)	41 (18)	22
Academics	9 (11)	15 (10)	24 (10)	33
Hospital doctors, specialists	7 (9)	0 (0)	7 (3)	100
Other (not specified)	-	5 (3)	5 (2)	-
**Location**				
Total number *	*86 (100)*	*143 (100)*	*229 (100)*	*38*
Metropolitan	67 (29)	113 (50)	180 (79)	37
Rural	19 (8)	29 (13)	48 (21)	40
Subgroups: South Australia	80 (35)	131 (57)	211 (93)	40
Interstate	6 (3)	11 (5)	17 (7)	35
Metropolitan South Australia	66 (29)	112 (49)	178 (78)	37
Rural South Australia	14 (6)	19 (8)	33 (15)	42
**Gender**				
Total number *	*88 (100)*	*141 (100)*	*229 (100)*	*38*
Female	68 (76)	99 (70)	167 (73)	41
Male	20 (23)	42 (30)	62 (27)	32

* depending on valid survey responses

Health service employees made up the largest group of health professionals (29% of 229 members) to join SARNet in 2002/03, but represented the group with the lowest response rate (17%). In contrast, few clinicians (7 hospital doctors, medical specialists) joined SARNet in 2002/03, but all returned the baseline survey. Other groups of health professionals with high response rates were general practitioners (54%) and allied health professionals (51%), while only 22% nurse members and 33% of academic members returned the survey (Figure 1b and Table 1).

Eighty respondents commented on their reason for joining the network and multiple answers for one individual were possible. The majority of respondents (57.5%) joined SARNet for the opportunity to network and share information, 35% wanted to build their research capacity, 24% were generally interested in primary health care, 21% sought mentoring support, 12.5% welcomed the opportunity to apply for funding of their research idea, 9% had heard about SARNet while doing research, and 4% hoped to combat their isolation by joining the network.

### Research categories

Eighty-eight of the 89 participants provided information to this question. About a third of the respondents (32%, n = 28) had managed their own research projects as a 'clinician researcher' and had participated in formal research training (category 3, manager/trainer). Twenty-seven percent (n = 24) had less experience in research but had been a member of a research team and had been involved in some research training and skills development (category 2, participant). Equal numbers of respondents (20.5%, n = 18) represented either novice researchers with little or no previous experience (category 1, non-participant) or experienced academics with or studying towards a doctoral degree (category 4, academic).

### Research experience and interest in up-skilling – the 'research spider'

Overall, most survey respondents indicated little to moderate experience (median score = 3 ± 1.1) in 7 out of the 10 skills depicted in the 'research spider'. The area of research in which most respondents (60%) reported the highest level of experience was 'finding relevant literature' (median score = 4 ± 1.1). In contrast, 60% of respondents indicated no or little experience in both 'publishing research' and 'applying for research funding' (median score = 2 ± 1.2).

In comparison, survey respondents were generally highly interested in developing their research skills in all areas. Over 60% of respondents indicated moderate to high interest in up-skilling in 9 out of 10 research areas, including 48.3% of respondents alone who scored 'high interest' in developing skills in 'analysis and interpretation of results' (median score = 4 ± 1.4). Lower overall interest was indicated for interest in up-skilling in 'finding relevant literature' (median score = 3 ± 1.5). Figure 2a illustrates the median research experience (red line) and interest in up-skilling (blue line) of all survey respondents.

Of particular interest to our capacity building program were the needs and skills of early career researchers. Accordingly, we analysed the responses from current category 1 researchers (non-participants) in the ten core research skill areas depicted in the 'research spider'. Figure 2b illustrates the median research experience (red) and interest in up-skilling (blue) of the category 1 practitioners. While the interest in up-skilling of category 1 practitioners was almost identical to the overall interest of all survey respondents, their research experience was expectedly lower compared to the median experience of all survey respondents.

### Research experience and interest in up-skilling by research category

**Figure 3:**
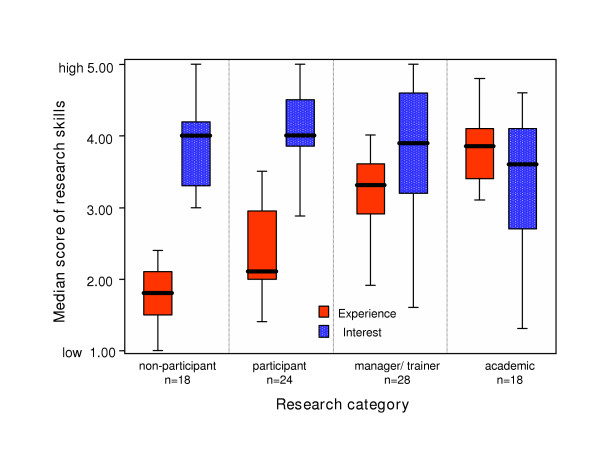
**Research experience and interest in skill development of survey respondents by research category**. The grouped median scores of individuals' experience or interest in ten research skill areas are plotted against four research categories. The ten research skill areas are 'writing a research protocol', 'using qualitative research methods', 'publishing research', 'writing and presenting a research report', 'analysing and interpreting results', 'using quantitative research methods', ' critically reviewing the literature', 'finding relevant literature', 'generating research ideas', and 'applying for research funding'. Median scores range between 1 (low experience or interest) to 5 (high experience or interest). Self-reported research skills for non-participants were generally low (median = 1.8 ± 0.4) and increased for each subsequent research category. The correlation between research skills and research category, both measures of research experience, is significant (red box plots, Spearman rank test, r = 0.82, p < 0.0005). Overall interest in development of skills in the ten core areas was high in all four research categories (blue patterned box plots; median = 3.6 to 4.0 ± 1.0).

Figure 3 illustrates the research experience of the ten skills depicted in the 'research spider' (red box plots) and the interest in up-skilling (blue box plots) in each of the four research categories. The self-reported skill levels (red) were significantly correlated with the self-reported participation level in research (Spearman rank test, r = 0.82, p < 0.0005). Overall interest in up-skilling (blue) was high in all four categories.

### Publication and funding record by research category

More than half of the respondents (54%) had never published any research, whereas two-thirds of published respondents (66%) had a least one publication in a peer-reviewed journal as the primary author. Not surprisingly, it was more likely that the respondent had published if (s)he had been more involved in research and therefore was associated with a higher research category. The correlation between publication record and research category was statistically significant (Gamma association, γ = 0.53, p < 0.0005).

A similar significant correlation was found between the number of research grants and the research category (Gamma association, γ = 0.62, p < 0.0005). About a quarter of the respondents (19 of 89) had received at least one research grant, with individual awards ranging from $2,000 to $337,500.

### Interest in research training, resources and networking activities

**Figure 4:**
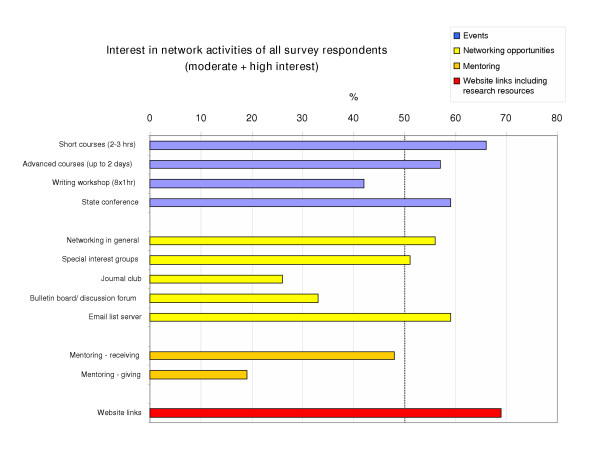
**Interest in network activities of all survey respondents**. Interest in network activities was generally high. Percentages of all survey respondents indicating moderate (score = 4) to high interest (score = 5) are illustrated for suggested research training events (blue), networking opportunities (yellow), mentoring (orange), and website links including research resources (red). The most popular activities with more than 60% of all respondents indicating moderate to high interest were short training courses of 2–3 hours and access to research related website links.

The level of interest in the majority of training, resources, and networking activities suggested in the survey was high across all categories. The median score for the level of interest was 3.5 on a five-point scale where 1=no interest and 5=high interest. Table 2 summarises the interest in network activities of respondents indicating moderate (score = 4) to high (score = 5) in each research category and overall. Figure 4 illustrates the interest in network activities of all survey respondents. While the number of respondents with these scores was small in some cases, more than half of all respondents indicated moderate to high interest in 7 out of 12 suggested network activities.

**Table 2:** Interest in network activities (moderate & high interest only)

	**Number (Percentage) of respondents indicating moderate & high interest**				
**Network activity**	**Category 1** **Non-participants**	**Category 2** **Participants**	**Category 3** **Managers/Trainers**	**Category 4** **Academics**	**Total** **All respondents**

	*n (%)*	*n (%)*	*n (%)*	*n (%)*	*n (%)*
***Total number in categories***	*18 (100)*	*24 (100)*	*28 (100)*	*18 (100)*	*88 (100)*
**Events**					
Short courses (2–3 hrs)	11 (61)	18 (75)	24 (86)	5 (28)	58 (66)
Advanced courses (up to 2 days)	9 (50)	15 (63)	14 (50)	12 (67)	50 (57)
Writing workshop (8 × 1 hr)	7 (39)	11 (46)	13 (46)	6 (33)	37 (42)
State conference	8 (44)	13 (54)	18 (64)	13 (72)	52 (59)
**Networking opportunities**					
Networking in general	9 (50)	13 (54)	14 (50)	13 (72)	49 (56)
Special interest groups	6 (33)	14 (58)	14 (50)	11 (61)	45 (51)
Journal club	4 (22)	8 (33)	5 (18)	6 (33)	23 (26)
Bulletin board/discussion forum	8 (44)	7 (29)	10 (36)	4 (22)	29 (33)
Email list server	8 (44)	13 (54)	19 (68)	12 (67)	52 (59)
Mentoring – receiving	6 (33)	14 (58)	12 (43)	10 (56)	42 (48)
Mentoring – giving	1 (6)	5 (21)	5 (18)	6 (33)	17 (19)
**Website links **e.g. training, funding, resources, primary health care journals	12 (67)	16 (67)	20 (71)	13 (72)	61 (69)

#### Research training events

Two thirds of the respondents showed moderate to high interest in short courses of 2–3 hours, and more than half were highly interested in advanced courses of up to 2 days and attending a state conference. Interest in a writing workshop was slightly lower (42% indicating moderate to high interest). The training format of 2–3 hour short courses was most popular for category 3 researchers, practitioners described as managers and trainers.

#### Networking opportunities

More than half of the respondents indicated moderate to high interest in networking in general, in participating in special interest groups, and staying connected via an email list server. Interest was lower in a journal club (26%) and in a bulletin board or discussion forum (33%). More than half of category 2 researchers (participants, n = 14 out of 24, 58%), and category 4 researchers (academics, n = 10 out of 18, 55%) were interested in receiving mentoring. A total of 17 respondents indicated moderate to high interest in mentoring others.

#### Website links

The majority of respondents (70%) were interested in all website links suggested in the survey, including training and funding opportunities, links to meetings and conferences, resources in evidence-based practice, and primary health care research and evaluation journals.

## Discussion

The survey results provided valuable information about early membership of the research network. Specifically, the survey illustrated the wide variety of backgrounds of respondents, existing capacity and interest, useful data about the motivation to join the network, and the likely training needs of participants. While capacity building using a bottom-up or top-down approach has been suggested by others [7], the results of our survey clearly indicate that a whole system approach more appropriately accommodates the capacity building needs of health care practitioners.

The Flinders PHCRED model of capacity building suggested differentiating between practitioners by separating them into four categories of research participation. Here, we show a significant correlation between the four research categories and participants' self-reported skill levels, thus validating the model's approach to this differentiation. For example, members of category 1, the non-participants, reported the most limited publication record and research funding, as well as the lowest experience in specific research skills. Categorising practitioners according to their involvement in research represents a practical way of planning capacity building activities designed to meet the needs of a particular group.

The "research spider" tool provided a simple and efficient way of representing respondents' existing skills, as previously reported by Smith et al. [6]. Our study built on this approach by also applying the 'research spider' tool to describe participants' interest in further development of their research skills. This enabled us to identify a gap between self-perceived skills and desired skills. Most respondents were interested in developing research skills, particularly if they were early-career researchers.

In addition to proposing a whole system approach, the Flinders PHCRED model for capacity building promotes networking and mentoring. This was validated by respondents indicating that networking and mentoring were significant factors in their decision to join SARNet. Similarly, access to peer support and individual support through mentoring, as well as knowledge skill training, were seen as key factors for joining research networks in Canada [8] and the UK [9,10].

We acknowledge that survey results are representative of only a proportion of members' background, skills and needs. However, these 89 members actively contributed to shaping network activities by taking part in the study. It could be speculated that some professional groups, such as the health service employees, had joined the network to observe upcoming PHCRED program activities, and were not necessarily actively seeking personal capacity building in research at this time. This hypothesis could explain, for example, the very low response rate of health service employees. In fact, networks like SARNet are dynamic in nature, offering multiple sites of learning at any time, which members can choose from in any combination according to their needs. Longer term analysis of the network and its members as well as longitudinal tracking of researchers at different levels will provide a measure of the success of various capacity building strategies and of the research network as a whole. One area of particular need in Australia is the support for practitioners in geographically isolated locations.

Our survey data allow capacity-building strategies to be designed to meet particular needs described by the members themselves. Training and other activities tailored to particular needs are more likely to be embraced by members and more likely to contribute to knowledge and capacity. Moreover, sustained membership of a network may result if members feel their capacity building needs are being met. The Flinders PHCRED program seeks feedback on its SARNet activities from members and undertakes structured evaluations of key components of network activities on a regular basis. The survey results presented here provided the basis for planning and development of network activities for its early years.

## Conclusion

There is increasing international interest and activity in building the research skills of primary care professionals. While many networks have been formed, many lack a systematic approach to understanding members' existing capacity and future needs. At the same time, other authors have drawn attention to the dearth of research on the theoretical basis of network operations [7]. Our results contribute to this theoretical knowledge by supporting the model for capacity building described above which groups Australian primary health care professionals into four research categories based on experience. Moreover, our survey has extended a previously reported tool, the 'research spider', to provide insights into the current research skills and future research interests and aspirations of a group of Australian primary health care practitioners. Evaluation of the quality of a network should include the degree of responsiveness to members' needs and also identify network achievements in the long-term.

## List of abbreviations

SARNet – South Australian Primary Health Care Research Network

PHCRED – Primary Health Care Research Evaluation Development program

## Competing interests

The author(s) declare that they have no competing interests.

## Authors' contributions

EAF and KMW conceptualized the study. KR was responsible for data analysis and illustration of results. All authors contributed to writing of the manuscript and approved the final version.

## Pre-publication history

The pre-publication history for this paper can be accessed here:



## Supplementary Material

Additional File 1 Aims and objectives for the SARNet research network Click here for file

Additional File 2 Categories of research experience Click here for file
