# Supplementary material for: Setting directions for capacity building in primary health care: a survey of a research network
Source: BMC Fam Pract. 2006 Feb 9;7:8. doi: 10.1186/1471-2296-7-8 (PMC1386681; doi:10.1186/1471-2296-7-8)
Supplement: Additional File 1 — Aims and objectives for the SARNet research network [file 1471-2296-7-8-S1.pdf]

Aims and objectives for the SARNet research network

To expand the pool of research-aware and research-oriented primary health care practitioners in South Australia and interstate by

1. Removing barriers and providing incentives for capacity building in research and evaluation to practitioners, students and consumers at all levels of research experience

2. Recognising the importance of diversity, in the interests, professional backgrounds, needs and learning styles of practitioners

3. Maintaining accessibility for Urban, Rural and Remote members

4. Recognising that research and evaluation skills are positive and necessary components of professional development and clinical practice in primary health care

5. Taking an integrated approach towards research and evaluation in primary health care to ensure, relevance of research and evaluation to improving clinical practice

6. Providing a forum to promote collaborative research

7. Developing research infrastructure to support the professional interests of the individual and to enhance corporate knowledge

8. Facilitating the integration of research and policy
