# Supplementary material for: Setting directions for capacity building in primary health care: a survey of a research network
Source: BMC Fam Pract. 2006 Feb 9;7:8. doi: 10.1186/1471-2296-7-8 (PMC1386681; doi:10.1186/1471-2296-7-8)
Supplement: Additional File 2 — Categories of research experience [file 1471-2296-7-8-S2.pdf]

Categories of research experience

Category 1 - Non-participants

Practitioners who have not participated in research, new researchers with little or no previous experience in research

Category 2 – Participants

Practitioners participating in research as part of a research team

Category 3 - Managers and trainers

Practitioners managing their own research project or in formal training to do so

Category 4 – Academics

Researchers with or leading towards a doctoral degree
